# A *pmoA*-based study reveals dominance of yet uncultured Type I methanotrophs in rhizospheres of an organically fertilized rice field in India

**DOI:** 10.1007/s13205-016-0453-3

**Published:** 2016-06-16

**Authors:** Pranitha S. Pandit, Dilip R. Ranade, Prashant K. Dhakephalkar, Monali C. Rahalkar

**Affiliations:** 1 MACS Agharkar Research Institute, G.G. Agarkar Road, Pune, Maharashtra 411004 India; 2 Microbial Culture Collection, NCCS, Sai-Trinity Building, Pashan, Pune, Maharashtra 411021 India

**Keywords:** Methanotrophs, Rice fields, Molecular approach, Clone libraries, *pmoA: gene for particulate methane monooxygenase subunit*

## Abstract

Rice fields are one of the important sources of anthropogenic methane. Methanotrophs can oxidize up to 30 % of the produced methane and thus have a pivotal environmental role in methane mitigation. India occupies the largest region under rice cultivation; however, most of the studies done on methanotrophic communities have focused on the Northern region. We studied methanotrophic community of a flooded, organically fertilized rice field using *pmoA* clone library approach. Organic rice fields impose a more serious threat as they produce more methane. *pmoA* gene is the main functional gene which is primarily used for taxonomical analysis of methanotrophs. Our results showed that the *pmoA* clone libraries from two growth stages of rice were dominated by *pmoA* sequences which were very distant from cultivated Type Ia methanotrophic genera (80–82 % nucleotide similarity) indicative of the presence of a putatively novel genus. We designated this group of clones as ‘rice field clones’ as this also includes many *pmoA* sequences originating from other rice fields. Thus, our current knowledge of methanotroph diversity from Indian rice fields has been expanded revealing that a substantial portion of methanotrophic diversity is unexplored.

**Electronic supplementary material:**

The online version of this article (doi:10.1007/s13205-016-0453-3) contains supplementary material, which is available to authorized users.

## Introduction

Global warming is the major environmental concern for the entire world. Methane is the second most important greenhouse gas and flooded rice fields are one of the major anthropogenic sources of atmospheric methane (Conrad 2009). Methanotrophs are a very important group of microorganisms which mediate biological methane oxidation mitigating methane and eventually have a major impact on controlling global warming. These bacteria act as environmental bio-filters and are known to be mainly present and active in the rhizospheres of rice fields. Methanotrophs dwelling in rice fields can oxidize up to 30 % of the produced methane (Shrestha et al. 2010). Cultivation of methanotrophs is challenging and the cultivable diversity does not always mirror the actual community present in the environment (Bussmann et al. 2004; Pandit et al. 2016). Molecular or culture independent studies give us a general picture of the diversity present and provide an important basis for cultivation based studies as these give us important hints on which communities dominate (Shrestha et al. 2010).

India has the largest area under rice cultivation in the world, ranks second in production and has been attributed to emit about 5–6 Tg methane per year (Manjunath et al. 2006; Yan et al. 2009). However, the actual methane emission values from this source are lower than the predicted values, i.e., 3.33 Tg/year (India Green House Gas Emissions 2007). One of the factors could be the presence of active and efficient methanotrophs in tropical Indian rice fields. Methanotroph communities present in rice fields under tropical climatic conditions could be entirely different compared to those present in temperate regions, e.g., China, Italy, etc. India is a tropical country which has an extremely hot summer followed by monsoonal rains (during June–September) and rice cultivation mainly takes place in this period. Under such conditions, rice fields remain flooded for a period of 3–4 months. Not much information is available on the diversity and activities of methanotrophs in lowland and flooded rice fields in India, especially from Southern or Central or Western regions. Recently, we have studied the culturable and unculturable diversity of a flooded rice field from Central Western region of India which was fertilized with inorganic fertilizer and planted with a traditional rice variety (Pandit et al. 2016). Application of organic fertilizers in rice fields poses more serious threat due to higher methane production (Dubey 2005; Jain et al. 2004) leading to higher methane emissions from these regions. Methanotrophic diversity from inorganically fertilized rice fields has been studied by many groups (Noll et al. 2008; Pandit et al. 2016; Vishwakarma and Dubey 2010a), but not much information is available on methanotroph diversity in organically fertilized rice fields. Organic amendments are common in Indian rice fields (Yan et al. 2009). In the present study, we focused on understanding the methanotroph community prevailing in rhizospheres of an organically fertilized, flooded rice field in Western Central India. As rhizospheres are hotspots for methane oxidation activities, we chose to study methanotroph communities from this microhabitat. We used *pmoA* gene which is the main functional gene found in methanotrophs and has been routinely used for analyzing the diversity and phylogeny in methanotrophs (Lüke et al. 2014; Luke et al. 2010; Ma et al. 2013). Recently, cutoff values for *pmoA* gene sequences have been established for demarcation at the species, genus and family level (Wen et al. 2016). In our present study, we have amplified and cloned *pmoA* gene using community DNA and analyzed the methanotrophic community. Sampling was done at two time points during the rice growth period to get a comprehensive picture of the community.

The rice field was naturally flooded during the rainy season, organically fertilized with vermi-compost prepared on site and the field is located at a village Kalbhorwadi, Urawade, near Pune city (18°37′N, 73°45′E), altitude 222 feet in Maharashtra State in India, planted with a local popular variety, ‘Indrayani’. This field has been used for rice cultivation for more than 40 years and has been organically fertilized throughout (personal communication with the farmer). Physico-chemical properties of the rice field soil are illustrated in Supplementary Table 1. Sampling was done at two time points, i.e., 43 days after transplantation (DAT) and at 84 DAT, representing the vegetative and reproductive stages of the plant, respectively. During vegetative stage, the field was flooded by rain water (water level was 7 cm), whereas when 84 DAT sample was taken, the field was moist but not flooded as the rains had subsided. Five randomly selected plants were uprooted with their intact root systems, placed in sterile plastic bags and brought to the laboratory within 2 h. Soil was shaken off from the roots by beating on a hard surface for 8–10 times and then the soil tightly attached to the roots was scraped into plastic tubes. A pooled sample was created by mixing equal aliquots of the soil, mixed and stored for DNA extraction at −20 °C. DNA extraction, PCR amplification, T-RFLP, cloning and analysis were done as described earlier (Pandit et al. 2016). Sequences from our previous study on an inorganically fertilized rice field (Pandit et al. 2016) and other closely related clones from other rice fields and habitats were included in the phylogenetic analysis. These *pmoA* sequences were phylogenetically analyzed using MEGA 6 software. Alignments were done using MAFFT online aligner (http://mafft.cbrc.jp/) and phylogenetic analysis was done using the maximum likelihood algorithms as implemented in MEGA using nucleotide data and 500 bootstrap replications. All sequences obtained in this study have been deposited in GenBank under accession numbers: KM995823.1–KM995836.1. Methane oxidation rates of soil samples were calculated as described earlier (Pandit et al. 2016).

### Type I uncultured methanotrophs dominate *pmoA* clone libraries

**Fig. 1:**
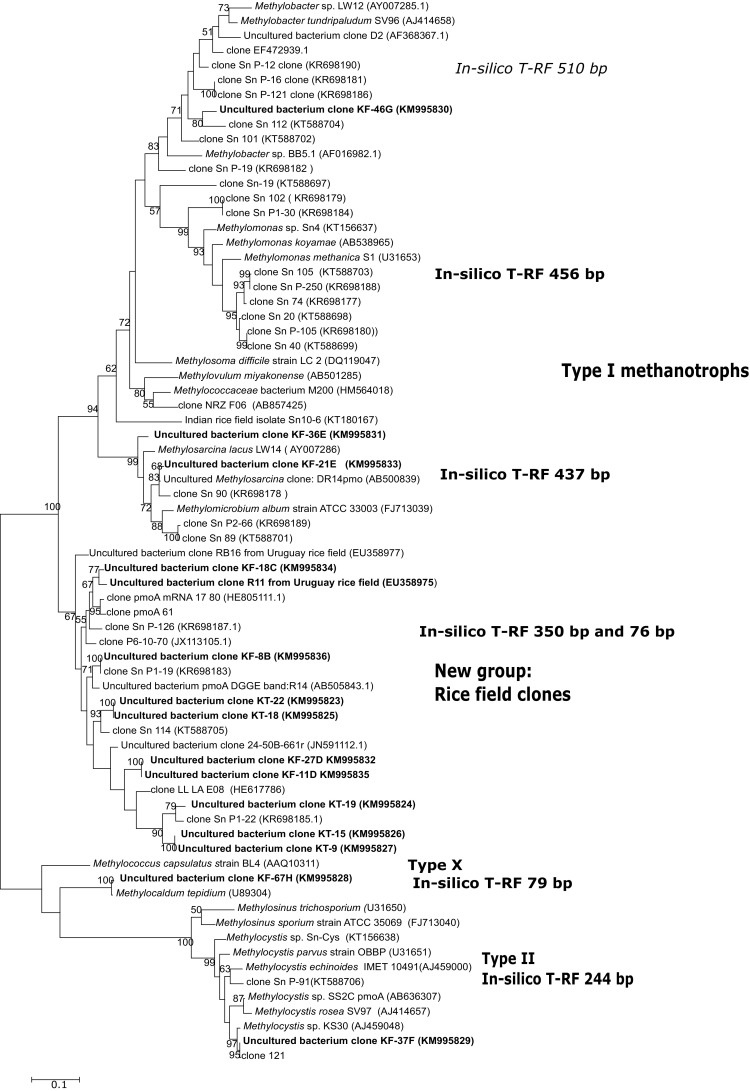
Phylogenetic tree of *pmoA* sequences obtained from clones and cultures from a rice field in Western India. KT represents Kalbhorwadi tillering stage, i.e., 43 DAT clones and KF represents Kalbhorwadi flowering stage, i.e., 84 DAT clones followed by clone number and RFLP group. National Center for Biotechnology Information accession numbers from other studies are given along with the names or clone numbers. The ‘rice field clones’ are indicated as a group. Clones prefixed with Sn are from our previous study (Pandit et al. 2016). The tree was constructed by the maximum likelihood method using Kimura 2 parameter and was based on 508 nucleotides and with 500 replications, the bootstrap values are shown. The *bar* represents 1 % divergence

The *pmoA* clone libraries showed a clear dominance of Type I methanotrophs in both the samples (43 DAT and 84 DAT). In case of 43 DAT sample, all the clones belonged to Type I methanotrophs after blast analysis, whereas in 84 DAT sample, 94 % methanotrophs belonged to Type I methanotrophs. All these *pmoA* clones from 43 DAT samples and 53 % *pmoA* clones from the 84 DAT sample were distantly related to Type I methanotrophs and showed 80–82 % sequence similarity with known genera (Tables 1, 2). These clones belonged to the clone groups B, C and C′ in case of 43 DAT and clone groups B, C and D from 84 DAT sample. The phylogenetic cultivated neighbors of these sequences were Type Ia methanotrophs of the genera, *Methylomicrobium, Methylosoma* and *Methylosarcina* showing 80–82 % similarity. When the clones were compared with uncultured *pmoA* sequences in the database, the clones showed hits with *pmoA* clones which were specifically originated from rice fields throughout the world including China, Uruguay, Italy and India. In phylogenetic analysis, all these sequences formed a unique group within Type I methanotrophs and the uniqueness of the branch was indicated by 100 % bootstrap value (Fig. 1). This group was designated as ‘rice field clone’ as it also included many clones originating from rice fields throughout the world (Fig. 1). Rest of the clones from 84 DAT sampling showed affiliation with other Type Ia methanotrophs and only 6 % clones were related to Type II methanotrophs and 4 % to Type Ib methanotrophs (*Methylococcus, Methylocaldum*), (Table 2). T-RFLP analysis of the 84 DAT sample confirmed the results obtained by clone library analysis (Supplementary Fig. 1). Major peaks were 76 and 350 bp which were the predicted in silico T-RFs of the rice field clones. Additionally, the other major peaks were at 437, 456 and 510 bp (Type I methanotrophs) and a minor peak was seen at 244 bp (Type II methanotrophs). The T-RFLP profile was similar to the profile obtained in our earlier study (Pandit et al. 2016) in terms of peak size; however, in the present study, higher peaks were obtained at 76 and 350 bp and comparatively lower peaks at 437 and 510 bp (Supplementary Fig. 1) in comparison to the profile obtained in our earlier study. The clone distribution in these two studies has been compared in Supplementary Table 2. It is clear that the clone group showing 350 and 76 bp in silico T-RF dominated the methanotrophic community, in the present study which is based on organically fertilized field. A substantial number of clones (16 %) from the previous study grouped within ‘rice field clones’ (clone groups D and E), Fig. 1 and (Pandit et al. 2016).

**Table 1:** *pmoA* clone library of rhizospheric sample 43 DAT

Clone number, RFLP type	Number of clones (total = 38)	Relative abundance (%)	Next relative (sequence identity) uncultured, % identity (nucleotide)	Next relative (sequence identity) cultured, type species, % identity (nucleotide)
KT-19 **C**	28	74	mRNA 17_3, (HE805103), 99 %	*Methylomicrobium album* (FJ713039), 80 %
KT-18, 22 **D**	7	18	Clone YT30-30 (KF823576.1), 93 %	*Methylomicrobium album* (FJ713039), 82 %
KT-9 **C′**	3	7	DGGE band (AB505834.1), 99 %	*Methylomicrobium album* (FJ713039), 80 %

* Clones belonging to ‘rice field group’ are shown in bold

**Table 2:** *pmoA* clone library of rhizospheric sample 84 DAT

Clone number, RFLP type	Number of clones (total = 49)	Relative abundance (%)	Next relative (sequence identity) uncultured, % identity (nucleotide)	Next relative (sequence identity) cultured, % identity (nucleotide)
KF-8, **B**	2	4	Clone (JX113105), 95 %	*Methylosoma difficile* (DQ119047) 82 %
KF-18, 42 **C**	17	35	Clone (HE805111), 96 %	*Methylomicrobium album (*FJ713039) 83 %
KF-11, 27 **D**	7	14	Clone (HE617786), 92 %	*Methylomicrobium album (*FJ713039) 80 %
KF-21, 36 E	13	27	Clone (AB500839), 99 %	*Methylomicrobium album (*FJ713039) 93 %
KF-37 F	3	6	Clone (AB845075), 98 %	*Methylocystis echinoides* (AJ459000), 95 %
KF-46 G	5	10	Clone (EF472939), 92 %	*Methylobacter tundripaludum* (AJ414658), 90 %
KF-67 H	2	4	-Cultured relative is closest-	*Methylocaldum tepidium* (MTU89304), 99 %

* Clones belonging to ‘rice field group’ are shown in bold

Type I methanotrophs have been shown to be associated with rice roots in various studies worldwide (Horz et al. 2001; Kolb et al. 2003; Ma et al. 2013). Horz et al. had used cloning, sequencing and T-RFLP approach for analyzing the methanotroph community from Vercelli, Italy (Horz et al. 2001). The dominant methanotrophs were affiliated to *Methylomonas*, *Methylobacter*, *Methylococcus* and to a novel Type I methanotroph sublineage distantly related to *Methylomicrobium*. Uncultured Type I methanotrophs similar to *Methylomicrobium album* have also been detected in Vercelli, Italy samples which were amended with Urea fertilizer (Noll et al. 2008), a RNA-based stable isotope probing study. Our study shows similar results, i.e., dominance of a Type I methanotrophs, albeit distantly related to *Methylomicrobium album*. Apart from rice fields, Type Ia methanotrophs have been demonstrated to be responsible for methane consumption in a variety of wetland ecosystems, including rice paddies, riparian wetlands, arctic wetlands, lake sediments (Bodelier et al. 2013). Our earlier study on methanotrophic communities from a flooded, inorganically fertilized rice field within 100 km distance had also revealed the dominance of Type I methanotrophs in rice rhizosphere community (Pandit et al. 2016). The clone groups and T-RFLP pattern found in the present study have been also found in several studies on rice field methanotrophs over the world, including Italy (Horz et al. 2001) and China (Ma et al. 2013). This also confirms that rice field methanotroph community shows a characteristic community composition throughout the world.

### Presence of a putative novel genus within Type I methanotrophs

Microbial molecular ecology of rice field methanotrophs has been almost exclusively studied using *pmoA* gene, the most used marker in molecular ecology studies of methanotrophs (McDonald et al. 2008). Phylogeny of *pmoA* gene largely corresponds to the phylogeny of 16S rRNA gene (McDonald et al. 2008; Wen et al. 2016). In a recent study, a systematic comparison of *pmoA* sequences and 16S rRNA gene sequences was done originating from 77 valid methanotroph species (Wen et al. 2016). Updated and weighted mean *pmoA* gene cutoff values based on the nucleotide level for genus level were established to be 82 % corresponding to 95 % 16S rRNA gene similarity (Wen et al. 2016). Looking at these cutoff values, there are clear indications of the presence of a novel genus with one or more species within the rice field clones group.

Methane oxidation rates were determined to measure the activity of the methanotrophs. It was observed that the reproductive stage (84 DAT) of the plant showed higher methane oxidation rate (1.43 µmol. methane/g dry weight/h) when compared to vegetative stage plant 43 DAT (0.30 µmol. methane/g dry weight/h), an observation similar to that done in Northern rice fields in India (Vishwakarma and Dubey 2010b).

To summarize, a unique methanotroph community inhabited the organically fertilized rice field in a tropical country, India and was composed of novel and yet uncultured Type I methanotrophs. There are clear indications of the presence of a putative novel genus within Type I methanotrophs, from this study. Recently, we have isolated one of the first methanotrophic strains from India which include a new putative genus and two putative novel species which indicates the presence of novel methanotrophs in Indian rice fields (Pandit et al. 2016). Our present study adds to our current knowledge about methanotroph diversity from Indian rice fields. Our future efforts would be targeted towards isolating and cultivating methanotrophs from such fields. This would help us in understanding the biochemistry and physiology of these fascinating groups of microorganisms, especially focusing on conservation of these unique taxa.

## Electronic supplementary material

Below is the link to the electronic supplementary material.

Supplementary material 1 (PDF 135 kb)
