# Supplementary figures and images for: A pmoA-based study reveals dominance of yet uncultured Type I methanotrophs in rhizospheres of an organically fertilized rice field in India
Source: 3 Biotech. 2016 Jun 16;6(2):135. doi: 10.1007/s13205-016-0453-3 (PMC4910840; doi:10.1007/s13205-016-0453-3)

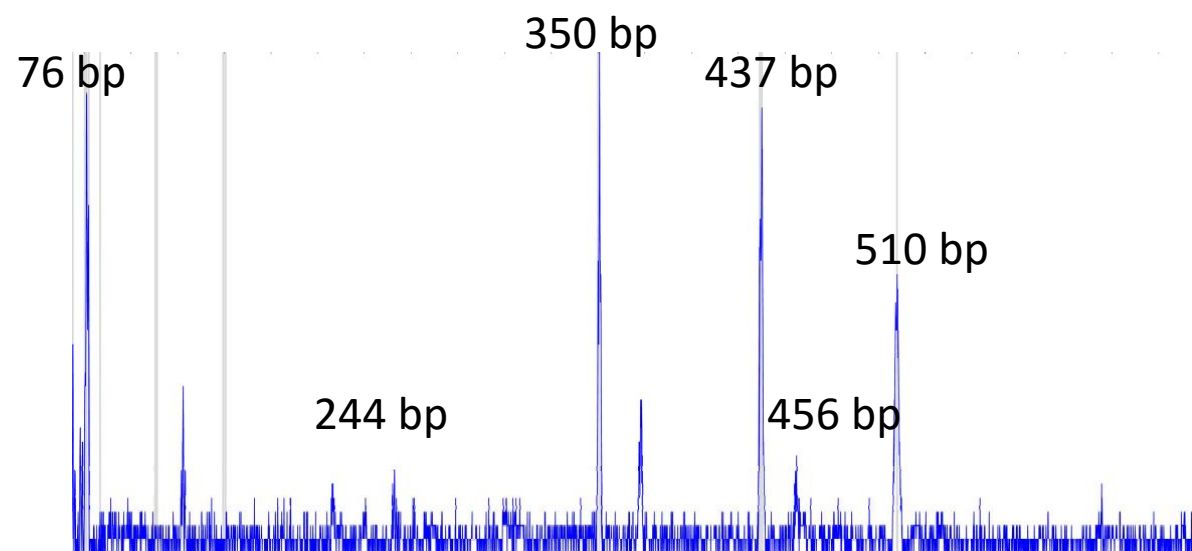

Supplement: Supplementary file 1 — Supplementary material 1 (PDF 135 kb) [file 13205_2016_453_MOESM1_ESM.pdf]
